# Expression of HIF-1α in medullary thyroid cancer identifies a subgroup with poor prognosis

**DOI:** 10.18632/oncotarget.15622

**Published:** 2017-02-22

**Authors:** Lutske Lodewijk, Paul van Diest, Petra van der Groep, Natalie ter Hoeve, Abbey Schepers, Johannes Morreau, Johannes Bonenkamp, Adriana van Engen - van Grunsven, Schelto Kruijff, Bettien van Hemel, Thera Links, Els Nieveen van Dijkum, Susanne van Eeden, Gerlof Valk, Inne Borel Rinkes, Menno Vriens

**Affiliations:** ^1^ University Medical Center Utrecht, Department of Surgery, 3584CX Utrecht, The Netherlands; ^2^ University Medical Center Utrecht, Department of Pathology, 3584CX Utrecht, The Netherlands; ^3^ University Medical Center Utrecht, Department of Endocrine Oncology, 3584CX Utrecht, The Netherlands; ^4^ Leiden University Medical Center, Department of Surgery, 2333ZA Leiden, The Netherlands; ^5^ Leiden University Medical Center, Department of Pathology, 2333ZA Leiden, The Netherlands; ^6^ Radboud University Medical Center, Department of Surgery, Nijmegen 6525GA, The Netherlands; ^7^ Radboud University Medical Center, Department of Pathology, Nijmegen 6525GA, The Netherlands; ^8^ University Medical Center Groningen, Department of Surgery, 9700 RB, Groningen, The Netherlands; ^9^ University Medical Center Groningen, Department of Pathology, 9700 RB, Groningen, The Netherlands; ^10^ University Medical Center Groningen, Department of Internal Medicine, 9700 RB, Groningen, The Netherlands; ^11^ Academic Medical Center Amsterdam, Department of Surgery, 1105 AZ, Amsterdam, The Netherlands; ^12^ Academic Medical Center Amsterdam, Department of Pathology, 1105 AZ, Amsterdam, The Netherlands

**Keywords:** medullary thyroid cancer, hypoxia inducible factor 1 alpha, immunohistochemistry, tissue microarray, oncology

## Abstract

**Background:**

Medullary thyroid cancer (MTC) comprises only 4% of all thyroid cancers and originates from the parafollicular C-cells. HIF-1α expression has been implied as an indicator of worse prognosis in various solid tumors. However, whether expression of HIF-1α is a prognosticator in MTC remained unclear. Our aim was to evaluate the prognostic value of HIF-1α in patients with MTC.

**Methods:**

All patients with MTC who were operated on between 1988 and 2014 in five tertiary referral centers in The Netherlands were included. A tissue microarray was constructed in which 111 primary tumors could be analyzed for expression of HIF-1α, CAIX, Glut-1, VEGF and CD31 and correlated with clinicopathologic variables and survival.

**Results:**

The mean age of patients was 46.3 years (SD 15.6), 59 (53.2%) were male. Of the 111 primary tumors, 49 (44.1%) were HIF-1α negative and 62 (55.9%) were HIF-1α positive. Positive HIF-1α expression was an independent negative indicator for progression free survival (PFS) in multivariate cox regression analysis (HR 3.1; 95% CI 1.3 – 7.3). Five-years survival decreased from 94.0% to 65.9% for the HIF-1α positive group (p=0.007). Even within the group of patients with TNM-stage IV disease, HIF-1α positivity was associated with a worse prognosis, shown by a decrease in 5-years survival of 88.0% to 49.3% (p=0.020).

**Conclusion:**

Expression of HIF-1α is strongly correlated with adverse prognosis of MTC. This could open up new ways for targeted systemic therapy of MTC.

## INTRODUCTION

Medullary thyroid cancer (MTC) accounts for 4% of all thyroid cancers and, in contrast to other forms of thyroid cancer, it arises from the parafollicular C-cells. It occurs either as a sporadic disease or in a hereditary context as a manifestation of the endocrine tumor syndrome Multiple Endocrine Neoplasia type 2 (MEN2) (20-25%) [1]. Hereditary tumors are characterized by activating mutations in the rearranged-during-transfection (RET) proto-oncogene. Sporadic MTCs harbor, in 50% of cases, an acquired mutation in the RET proto-oncogene. About 45% of all patients present with advanced disease (stage III-IV), which has a 10-year survival rate of 71% and 21%, respectively [2].

Currently, clinical variables such as age, extent of the primary tumor, lymph node metastases and distant metastases have been identified as prognostic factors [2]. No further stratification for stage III-IV patients is available, while prognosis within these groups can vary widely [3]. To set the indication for follow-up or adjuvant treatment, patients with poor prognosis need to be identified, especially within the subgroup of stage III-IV patients.

Hypoxia inducible factor-1 (HIF-1) is the key regulator of the hypoxia response. HIF-1 is a protein complex composed of two subunits; constitutive HIF-1β and oxygen-sensitive HIF-1α. HIF-1α can either be upregulated due to oncogenic signaling or as a response to tumor hypoxia. Under normal conditions the HIF-1α subunit is degraded by the ubiquitin-proteasome pathway. Under hypoxia or as a result of oncogenic signaling, degradation is inhibited resulting in its accumulation, subsequent binding to HIF-1β, translocation to the nucleus and activation of downstream signaling pathways by binding to hypoxia responsive elements in the promoters of target genes [4–9]. Increased expression of HIF-1α in tumor cells, whether induced by hypoxia or by aberrant oncogenic signaling, actively drives tumor growth and progression by regulating the expression of crucial target genes such as vascular endothelial growth factor (VEGF), carbonic anhydrase IX (CAIX) and glucose transporter 1 (Glut-1) [8, 10].

In this regard, the importance of HIF-1α and its downstream targets has been investigated in various solid tumors, and the correlation of high HIF-1α expression with poor prognosis has been well established [10]. In sporadic MTC, expression of HIF-1α has been reported in 89% of tumors, and associated with clinical features, as lymph node positivity, higher T-stage and extrathyroidal extension, which are known to adversely affect prognosis [11]. However, due to the lack of survival data, prognostic value of HIF-1α in MTC could not be determined. Our aim was therefore to investigate the prognostic value of HIF-1α expression in MTC.

## RESULTS

### Clinicopathologic variables

The mean age of the 111 patients was 46.3 years (SD 15.6), 59 (53.2%) were male. Sixty-five patients (60.2%) had a sporadic medullary thyroid carcinoma, 39 patients (36.1%) had MEN2a and 4 patients (3.7%) had MEN2b, from 3 patients the RET-mutation status was unknown. Fifteen patients presented with stage 1 (13.5%), 26 with stage II (23.4%), 17 (15.3%) with stage III and 53 (47.7%) with stage IV disease. Median tumor size was 25.6 mm (IQR 25; minimum 2 mm; maximum 80 mm) and 79 (63.1%) patients presented with lymph node metastases. Mean follow-up was 79.2 months (SD 60.6).

### Association between HIF-1α expression and clinicopathologic variables

Forty-nine (44.1%) patients were HIF-1α negative and 62 (55.9%) HIF-1α positive. In univariate analysis, age, gender, heritability, stage, tumor size, lymph node metastases, disease status, microvessel density (MVD) and presence of necrosis or angioinvasion did not differ significantly between HIF-1α negative and positive groups (Table 1). For overall survival (OS) and PFS a significant association with HIF-1α status was found. Forty-six (93.9%) patients in the HIF-1α negative versus 49 (79.0%) patients in the HIF-1α positive group survived (p = 0.03). Forty-two (85.7%) patients in the HIF-1α negative versus 38 (61.3%) patients in the HIF-1α positive group did not show progression, i.e. they did not develop distant metastasis (p = 0.01). Grade of desmoplasia correlated significantly with HIF-1α expression, in the moderate to severe group 40 patients (66.7%) showed HIF-1α positivity (p = 0.00). CAIX, Glut-1 and VEGF showed a positive correlation with HIF-1α, with an OR of 1.5, 4.2 and 1.4 respectively, however, this correlation was not significant (Table 1).

**Table 1:** Clinicopathological characteristics of all patients stratified by HIF-1α status

	HIF-1α neg		HIF-1α pos		
	N=49		N=62		
**Mean age in years (SD)**	45.0	(14.1)	47.2	(16.7)	0.46
**Gender**					0.71
**Male (%)**	25	(51.0)	34	(54.8)	
**Heritability**					0.33
**Sporadic (%)**	33	(67.3)	32	(54.2)	
**MEN2a/b (%)**	16	(28.6)	27	(42.4)	
**Stage**					0.23
**I – III (%)**	28	(10.2)	30	(16.1)	
**IV (%)**	21	(42.9)	32	(51.6)	
**Size mean in mm (SD)**	26.1	(15.2)	25.1	(15.5)	0.75
< **20mm (%)**	20	(40.8)	22	(38.6)	0.84
**≥ 20mm (%)**	29	(59.2)	35	(61.4)	
**Lymph node metastasis**	28	(57.1)	42	(67.7)	0.32
**Overall Survival**	46	(93.9)	49	(79.0)	0.03
**Progression Free Survival**	42	(85.7)	38	(61.3)	0.01
**Disease status**					1.00
**Normal CEA/calcitonin (%)**	17	(37.8)	22	(36.7)	
**Elevated CEA/calcitonin (%)**	28	(62.2)	38	(63.3)	
**Presence of necrosis**	3	(6.2)	6	(10.0)	0.73
**Presence of angioinvasion**	4	(8.3)	6	(10.0)	1.00
**Presence of desmoplasia**					0.00
**None – some**	32	(66.7)	20	(33.3)	
**Moderate – severe**	16	(33.3)	40	(66.7)	
**CAIX**					0.34
**Neg (%)**	29	(59.2)	30	(49.2)	
**Pos (%)**	20	(40.8)	31	(50.8)	
**Glut-1**					0.23
**Neg (%)**	48	(98.0)	57	(91.9)	
**Pos (%)**	1	(2.0)	5	(8.1)	
**MVD mean vessels/core**	15.9	(9.7)	13.2	(5.8)	0.10
< **14 vessels/core**	22	(48.9)	35	(58.3)	0.43
**≥ 14 vessels/core**	23	(51.1)	25	(41.7)	
**VEGF**					0.41
**Neg (%)**	18	(41.9)	20	(33.3)	
**Pos (%)**	25	(58.1)	40	(66.7)	

Abbreviations: SD = Standard Deviation; MEN2a/b = Multiple Endocrine Neoplasia type 2a/b; CAIX = carbonic anhydrase IX; Glut-1 = glucose transporter 1.

### Prognostic value

**Figure 1:**
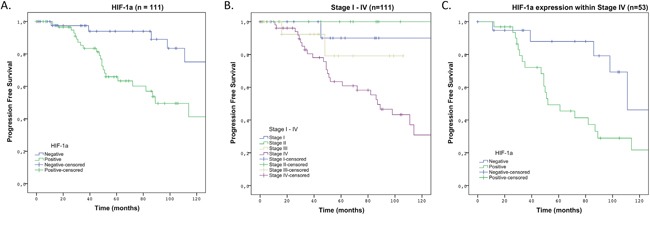
10-years PFS in Kaplan-Meier survival curve in patients with HIF-1α positive MTC compared to patients with HIF-1α negative MTC **(A)** Analyses over total group of 111 patients comparing HIF-1α positive versus HIF-1α negative MTC. **(B)** Analyses over total group comparing TNM-stage I-IV. **(C)** Analyses over subpopulation of TNM-stage IV patients comparing HIF-1α positive versus HIF-1α negative MTC.

To verify which variables were associated with PFS and OS a univariate survival analysis was performed (Table 2). For PFS a significant association was found for TNM-stage, presence of lymph node metastasis, heritability, HIF-1α, necrosis and Glut-1. For OS an association was found for heritability, disease status, necrosis, HIF-1α and VEGF. Our total number of events, patients that developed metastases and/or died, (n = 33) restricted us in the number of variables for multivariate analysis; therefore we studied in a Cox regression analysis the association of HIF-1α, TNM-stage and heritability on PFS. The variable lymph node metastasis was left out of the Cox regression analysis since this is also taken into account by the TNM-stage; disease status was left out since this is predominantly base on postoperative CEA/calcitonin measurements which are of value to evaluate over time to show tumor progression, but not as a single postoperative measurement, Glut-1 was left out as a down-stream effector of HIF-1α; necrosis was left out since only 9 patients showed necrosis, and with a prevalence of only 8.1% it is not suitable as a prognostic factor. In this multivariate analysis, we found that HIF-1α positivity, TNM-stage IV and sporadic MTC were all significantly and independently correlated to PFS (Table 3). HIF-1α positivity increased the risk of developing distant metastases with a HR of 3.1 (95% CI 1.3 – 7.3) per year. Hazard-ratios were 6.8 (95% CI 2.0 – 22.9) and 0.4 (95% CI 0.16 – 0.95) for Stage IV disease and MEN2a/MEN2b syndromes respectively. No violations of the proportional hazards assumption were found. In Figure 1A and 1B Kaplan-Meier curves visualize the prognostic effect of HIF-1α positivity and of Stage IV disease. In Figure 1C we show that also within TNM-stage IV disease, HIF-1α is able to identify patients with a less favorable outcome. Five-year survival rates were 55% for the HIF-1α positive group versus 95% for the HIF-1α negative group. Within the TNM-stage IV group survival decreased to 35% for the HIF-1α positive group versus 90% for the HIF-1α negative group.

**Table 2:** Univariate Kaplan-Meier survival analysis on PFS and OS

	Progression Free Survival				Overall Survival			
	N	PF (N)	PF (%)	p- value	N	OS (N)	OS (%)	p- value
**Stage**				0.00				0.14
**I-III**	56	53	94.6		53	48	90.6	
**IV**	52	24	46.2		49	38	77.6	
**Gender**				0.09				0.62
**Male**	56	36	64.2		53	44	83.0	
**Female**	52	11	78.8		49	42	85.7	
**Tumor size**				0.54				0.71
< **20mm**	40	33	82.5		39	34	87.2	
**≥ 20mm**	63	42	68.3		59	50	84.7	
**Lymph node metastasis**				0.00				0.09
**Yes**	39	37	94.9		37	34	91.9	
**No**	69	40	68.4		65	52	80.0	
**Heritability**				0.00				0.01
**Sporadic**	62	40	64.5		57	45	78.9	
**MEN2a/b**	43	36	83.7		42	39	92.9	
**HIF-1α**				0.01				0.04
**Neg**	47	40	85.1		44	41	93.2	
**Pos**	61	37	60.7		58	45	77.6	
**Disease status**				0.00				0.05
**Normal CEA/calcitonin**	39	37	94.9		39	37	94.9	
**Elevated CEA/calcitonin**	66	37	56.1		62	48	77.4	
**Necrosis**				0.01				0.01
**Neg**	96	25	74.0		92	79	85.9	
**Pos**	9	6	33.3		7	4	57.1	
**Angioinvasion**				0.18				0.67
**Neg**	95	26	72.6		90	75	83.3	
**Pos**	10	5	50.0		9	8	88.9	
**Desmoplasia**				0.16				0.30
**None – Some**	51	10	80.4		49	44	89.8	
**Moderate – Severe**	54	21	61.1		50	39	78.0	
**CAIX**				0.69				0.49
**Neg**	59	45	76.3		58	51	87.9	
**Pos**	48	32	66.7		43	34	79.1	
**Glut-1**				0.02				0.51
**Neg**	102	74	72.5		97	81	83.5	
**Pos**	6	3	50.0		5	5	100.0	
**MVD**				0.53				0.74
< **14**	58	39	67.2		54	45	83.3	
**≥ 14**	45	36	80.0		43	38	88.4	
**VEGF**				0.37				0.01
**Neg**	37	26	70.3		34	32	94.1	
**Pos**	64	48	75.0		61	49	80.3	

Abbreviations: N = number; PF = progression free; OS = overall survival; MEN2a/b = Multiple Endocrine Neoplasia type 2a/b; CAIX = carbonic anhydrase IX; Glut-1 = glucose transporter 1.

**Table 3:** Multivariate cox-regression analysis on PFS

	Events/patients	Adjusted Hazard Ratio (95% CI)	p-value
**HIF-1α**			0.01
**Negative**	7/49	1	
**Positive**	24/62	3.1 (1.3 – 7.3)	
**Stage**			0.00
**I-III**	3/56	1	
**IV**	28/52	6.8 (2.0 – 22.9)	
**Heritability**			0.04
**Sporadic**	22/62	1	
**MEN2a/b**	7/43	0.4 (0.16 – 0.95)	

Abbreviations: HIF-1α = Hypoxia Inducible Factor 1α; MEN2a/b = Multiple Endocrine Neoplasia type 2a/b.

## DISCUSSION

This study shows that HIF-1α is associated with the disease course of MTC and could, therefore, be a valuable prognostic marker. Survival decreases significantly when HIF-1α was expressed, with five-year survival rates of 95% for HIF-1α positive MTC versus 55% for HIF-1α negative MTC. As almost half of patients with MTC present with stage III-IV disease, indicating that the tumor already spread to either lymph nodes in the neck or to distant organs such as lung, liver or bones, a marker that can distinguish patients with a good survival within this group is warranted. Until now, prognostic markers in MTC that were identified, seemed to be of little clinical relevance, especially within the group with distant metastasis [12–16]. In contrast, HIF-1α did discriminate between less and more favorable prognosis within the group with TNM-stage IV in the present study.

Besides TNM-stage, CEA and calcitonin are well known valuable prognostic factors in MTC. In this study only direct postoperative measurements were included. CEA and calcitonin have the greatest prognostic value in the course of disease, i.e. faster doubling times give a worse prognosis [17, 18]. This means CEA and calcitonin are able to monitor tumor progression over time. However, we were more interested in pre- or postoperatively identifying patients who have a worse prognosis, thus identifying those patients who are likely to have short doubling times of CEA and calcitonin later on, to be able to prevent extensive tumor progression.

Koperek et. al. investigated HIF-1α expression in tumor tissue from 100 patients with sporadic MTC. They found expression of HIF-1α in 51% of cases, using the same threshold for HIF-1α positivity as we did. In line with their findings, our percentage of HIF-1α positivity was 56%. They did not show survival data, however correlations were found with variables that are known to correlate to poor prognosis i.e. grade of desmoplasia, T-stage and lymph node metastasis [11]. Our data confirmed their findings, since a significant correlation was found between HIF-1α expression and TNM-stage, lymph node metastasis and grade of desmoplasia.

Our study demonstrates that high HIF-1α expression in the primary MTC specimen is associated with poor outcome, especially in patients with advanced stage. Patients who are in need of a more intense follow-up can be identified, and this might have consequences for adjuvant therapeutic regimens. The prognostic value of HIF-1α in MTC is in line with findings in numerous studies performed in other cancer-types like; bladder -, breast -, cervical -, colorectal -, gastric -, head and neck -, non-small cell lung -, ovarian -, pancreatic -, prostate cancer and glioblastoma, glioma, melanoma, gastrointestinal stromal tumor and renal cell carcinoma. This has led to the generally accepted idea that increased expression of HIF-1α actively drives tumor growth and progression by regulating the expression of important target genes [10].

For CAIX expression only a weak-positive correlation was found with HIF-1α positivity. The relationship between CAIX and HIF-1α is complicated, since HIF-1α can also be upregulated by oxygen independent factors. This may effect the extent of their co-localization. Furthermore, the tissues examined are a 'snap-shot' in time, due to the very swift and dynamic kinetics of HIF-1α and the slower transcription of CAIX co-localization can be missed [19]. For Glut-1 a strong correlation (OR 4.2) was found, the lack of significance might be best explained by the relative low number of Glut-1 positive tumors (5.4%). Cytoplasmic staining of VEGF was seen in all MTCs however in a varying degree, we interpreted the VEGF staining as positive when there was a moderate to strong reactivity [20, 21]. No significant correlation was found with HIF-1α, however VEGF is known to be upregulated by RET mutations and sporadic RET mutations are not included in our data [20]. However, we did find that patients with high VEGF expression had a significant shorter overall survival [22].

Our results also raise interest in targeting HIF-1α in MTC. Targeting hypoxia is challenging. There are in short two main approaches: bioreductive prodrugs and inhibition of molecular targets in hypoxic cells. The main advantage of the bioreductive prodrugs is the ability to, selectively, attack hypoxic cells, resulting in a low toxicity profile. Therefore, they have a greater opportunity for a combination with current standards of therapy; Vandetanib and Cabozantinib [23]. However, the present study is merely a prognostic one, and further *in vitro* - and *in vivo* studies should be developed to investigate the role of bioreductive prodrugs in combination with tyrosine kinase inhibitors in MTC.

One of the strengths of this study is the fact that we combine immunohistochemical data with clinical endpoints such as survival or the occurrence of distant metastases in a relatively large sample size. Furthermore, since MTC is in most cases a relatively low-proliferating tumor, event-rates are low and a long follow-up is needed to detect them. Our follow-up is long (mean 70.2 months; SD 60.6) and we used PFS to increase the total number of events. One of the limitations is that immunohistochemistry is inherently a more qualitative than quantitative method. Furthermore, one might argue that due to heterogeneity of the HIF-1α, CAIX and Glut-1 staining pattern the use of tissue microarrays is suboptimal. However, studies investigating concordance between whole slide analysis and TMA results found good concordance in general [24]. Moreover, tissue microarrays are described as the standard for the validation of prognostic biomarkers [25, 26] and have been used in studies investigating the same proteins [27–30]. Further limitations are merely due to its retrospective character and the low incidence of MTC. A total of 5 tertiary referral centers have participated and patients over almost 3 decades have been included. To overcome this we limited our analyses to variables least subject to treatment changes overtime or interinstitutional differences.

In summary, HIF-1α overexpression is a prognostic biomarker in MTC indicating a worse prognosis, particularly, in the subpopulation with TNM-stage IV. Thus, HIF-1α may be clinically useful to identify patients in need of more intense follow-up or adjuvant therapy, and may provide an interesting therapeutic target in MTC.

## MATERIALS AND METHODS

### Patients

Patients who underwent surgery between 1988 and 2014 for MTC were identified from the pathology databases of Leiden University Medical Center (LUMC), Amsterdam Medical Center (AMC), Radboud University Medical Center (RadboudUMC), University Medical Center Groningen (UMCG) and University Medical Center Utrecht (UMCU), The Netherlands (all tertiary referral centers). Formalin fixed paraffin embedded (FFPE) tissues were collected from the pathology archives. In total 111 patients were identified from who primary tumor tissue was available for inclusion in the tissue microarray (TMA).

Whole slides were scored for necrosis, angioinvasion and desmoplasia. Necrosis and angioinvasion were scored as absent or present and desmoplasia as negative, some, moderate or severe. These scorings were performed on the same FFPE blocks that were used for the construction of the TMA.

Clinical and pathological patient information was retrieved from patient files in all five centers. All MEN2 diagnoses were confirmed by germline mutation analysis, sporadic patients were either patients with negative germline mutation analysis or with a negative family history. Microscopic positive resection margins were considered as part of the T-stage and not included as a separate variable. Disease status was based on postoperative calcitonin and CEA measurements; this was scored as a dichotomous variable. Since we included patients from five centers over almost three decades different assays were used for CEA and calcitonin measurements, therefore making it impossible to compare exact values. An elevation in CEA or calcitonin was interpreted as persistent disease, an CEA or calcitonin within normal range was interpreted as cured. Only postoperative CEA and calcitonin measurements were taken into account. Due to the fact that CEA and calcitonin measurements were performed in five centers over almost three decades and different assays were used, doubling times could not reliably be assessed. This study was performed according to national guidelines with respect to the use of leftover tissue [31] and approval for this study was obtained from the Institutional Review Board of the UMCU.

### Construction of tissue microarray

The TMA was developed on the TMA machine (TMA grand master, 3D Histec, Budapest, Hungary). Three cores of 0.6 mm were punched from FFPE blocks of the primary tumor. To assure that cores were punched from tumor areas, cell rich areas were marked on H&E slides by a pathologist (PJvD), scanned, and marks were manually circled with the TMA software (3D Histech). In this manner cell rich punches were automatically inserted into the recipient block.

### Immunohistochemistry

After deparaffinization and rehydration, endogenous peroxidase was blocked in a buffer solution containing 0.3%hydrogen peroxidase for 15 minutes. For HIF-1α antigen retrieval was performed using EDTA buffer, pH = 9.0, at boiling temperature for 20 minutes. A cooling period of 30 minutes preceded the incubation of the slides with protein block (Novolink Max Polymer detection system, ready to use, Novocastra Laboratories Ltd, Newcastle Upon Tyne, UK) for 5 minutes at room temperature. Incubation of the slides with the HIF-1α mouse monoclonal (BD Biosciences, Pharmingen, Lexington, MA, USA), was done at a dilution of 1:50 overnight at 4°C. For detection a polymer (Novolink Max Polymer detection system, ready to use, post primary for 30 minutes and Novolink Polymer for 30 minutes) was used and developed with diaminobenzidine (5 minutes, Novolink Polymer detection system). For Glut-1, CAIX and VEGF-A, downstream targets of HIF-1α, antigen retrieval was performed in citrate buffer, pH = 6.0, for 20 minutes at boiling temperature. For Glut-1 and CAIX, a cooling period of 30 minutes preceded the incubation (60 minutes at room temperature) with the primary antibodies. Polyclonal primary antibodies used were Glut-1 (1:200, DAKO, Santa Clara, USA) and CAIX (1:1000, Abcam, Cambridge Science Park, Cambridge, UK). For VEGF-A, a cooling period of 30 minutes preceded the incubation of the slides with the VEGF-A rabbit polyclonal antibody (0.2 μg/mL, RB-9031, ThermoFisher, Fremont, USA)[32]. For detection of the primary antibodies a poly HRP anti- Mouse/Rabbit/Rat IgG (Brightvision ready to use, 30 minutes. ImmunoLogic, Duiven, The Netherlands) was used. All slides were developed with diaminobenzidine (10 minutes) followed by hematoxylin counterstaining. Before the slides were mounted, all sections were dehydrated in alcohol and xylene. Positive and negative controls were used throughout.

To calculate microvessel density (MVD) CD31 immunohistochemistry was performed on the automatic system (BenchMark ULTRA, Ventana Medical System, Tucson, Arizona). The CD31 mouse monoclonal antibody, clone JC70A (1:100, DAKO, Santa Clara, USA).

### Scoring of immunohistochemistry

**Figure 2:**
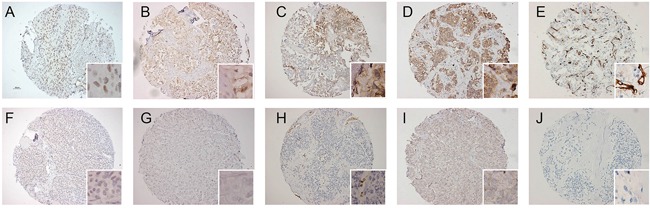
Representative examples of immunohistochemical staining pattern for HIF-1α, CAIX, Glut-1, VEGF and MVD **(A)** HIF-1α nuclear staining pattern in 50% of cells, **(B)** focal membranous CAIX immunoreactivity, **(C)** focal membranous Glut-1 immunoreactivity, **(D)** strong cytoplasmic VEGF immunoreactivity, **(E)** high MVD shown by CD31 immunoreactivity, **(F)** absent HIF-1α staining, **(G)** absent CAIX staining, **(H)** absent Glut-1 in tumor cells, with control positivity in red blood cells, **(I)** weak VEGF immunoreactivity, **(J)** MVD is 0 shown by absence of CD31 immunoreactivity.

All TMA slides were scored by an experienced pathologist (PJvD) and an experienced researcher (LL), when there was inconsistency between both a 2^nd^ experienced pathologist was consulted. For HIF-1α the percentage of positive nuclei per core was scored as an absolute number. For statistical analysis this was transformed in a dichotomous variable, which was positive when in either one of the three cores ≥1 percent of nuclei were positive as previously used [8, 11, 27]. Glut-1 and CAIX were scored as absent, cytoplasmic or membranous for each core separately. For statistical analysis only membranous staining was taken into account, when this was present for either one of the cores the tumor was considered positive [8]. VEGF-A was scored as absent (0), weak (1), moderate (2) or strong (3) for each core separately. For statistical analysis the average score over three cores was calculated and when this was ≥2 it was considered positive. MVD was calculated by the average number of CD31 positive vessels per core. For the dichotomous variable of CD31, a cut-off of 14 (mean) was chosen. Representative scores of all immunostainings are shown in Figure 2.

### Statistical analysis

Categorical data were summarized with frequencies and percentages, and continuous data were summarized with medians and ranges. Progression was defined as development of distant metastases or dead. This excluded development of lymph node metastases or elevation in CEA/calcitonin. We chose this definition since MTC is an incurable disease when distant metastases occur. Progression-free survival (PFS) was therefore defined as the time to development of distant metastases or dead. To increase the power of the statistical analysis categorical data were recoded into dichotomous variables. Stage I, II, III and IV was recoded into stage I - III and stage IV; hereditability was recoded in either sporadic or hereditable; grade of desmoplasia was recoded in none - some and moderate - severe.

The chi-square test was used to assess associations between the dichotomous variables, the Student's t-test was used to test for differences between continuous variables. Kaplan-Meier survival curves were plotted, and univariate survival analysis was performed and the log-rank test was used to calculate significance. Multivariate Cox-regression analysis was performed and Hazard Ratios (HR) of clinicopathologic characteristics on PFS were calculated. Violations of the proportional hazards assumption were tested by the log minus log plot and by adding a time dependent covariate. All reported p-values were two sided. Analysis was performed using SPSS version 22.0 software (SPSS, Inc., Chicago, IL, USA).
